# Allele-specific RT-PCR for the rapid detection of recurrent *SLC12A3* mutations for Gitelman syndrome

**DOI:** 10.1038/s41525-021-00230-8

**Published:** 2021-08-13

**Authors:** Ming-Tso Yan, Sung-Sen Yang, Min-Hua Tseng, Chih-Jen Cheng, Jeng-Daw Tsai, Chih-Chien Sung, Yu-Juei Hsu, Shih-Hua Lin

**Affiliations:** 1 Division of Nephrology, Department of Medicine, Cathay General Hospital, School of Medicine, Fu-Jen Catholic University, Taipei, Taiwan; 2 Graduate Institute of Medical Sciences, National Defense Medical Center, Taipei, Taiwan; 3 Division of Nephrology, Department of Medicine, Tri-Service General Hospital, Taipei, Taiwan; 4 Division of Pediatric Nephrology, Department of Pediatrics, Chang Gung Memorial Hospital, Taoyuan, Taiwan; 5 Division of Nephrology, Department of Pediatrics, MacKay Memorial Hospital, Taipei, Taiwan

**Keywords:** Genetic predisposition to disease, Genetic counselling

## Abstract

Recurrent mutations in the *SLC12A3* gene responsible for autosomal recessive Gitelman syndrome (GS) are frequently reported, but the exact prevalence is unknown. The rapid detection of recurrent *SLC12A3* mutations may help in the early diagnosis of GS. This study was aimed to investigate the prevalence of recurrent *SLC12A3* mutations in a Taiwan cohort of GS families and develop a simple and rapid method to detect recurrent *SLC12A3* mutations. One hundred and thirty independent Taiwan families with genetically confirmed GS were consecutively enrolled to define recurrent *SLC12A3* mutations and determine their prevalence. Using TaqMan probe-based real-time polymerase chain reaction, we designed a mutation detection plate with all recurrent mutations. We validated this mutation detection plate and tested its feasibility in newly diagnosed GS patients. A total of 57 mutations in the *SLC12A3* gene were identified and 22 including 2 deep intronic mutations were recurrent mutations consisting of 87.1% (242/278, 18 triple) of all allelic mutations. The recurrent mutation-based TaqMan assays were fully validated with excellent sensitivity and specificity in genetically diagnosed GS patients and healthy subjects. In clinical validation, recurrent mutations were recognized in 92.0% of allelic mutations from 12 GS patients within 4 h and all were confirmed by direct sequencing. Recurrent *SLC12A3* mutations are very common in Taiwan GS patients and can be rapidly identified by this recurrent mutation-based *SLC12A3* mutation plate.

## Introduction

Gitelman syndrome (GS) is characterized by persistent renal salt wasting, chronic hypokalemic metabolic alkalosis with renal potassium (K^+^) wasting, hypomagnesemia with inappropriate renal magnesium (Mg^2+^) loss, and hypocalciuria. Clinical manifestations of GS vary dramatically from asymptomatic, fatigue, dizziness, numbness, paresthesia, joint pain (pseudogout and chondrocalcinosis), and neuromuscular weakness to paralysis or fatal arrhythmia^1–4^. Patients with GS were also found to carry an increased risk of the development of chronic kidney disease (CKD) and type 2 diabetes mellitus^5^.

GS is inherited as autosomal recessive renal tubulopathy caused by inactivating mutations in the *SLC12A3* gene encoding thiazide-sensitive sodium chloride cotransporter (NCC) on the apical membrane of distal convoluted tubule^6,7^. Given a much higher prevalence of GS carrier (1–4%), GS may be the most common inherited salt-losing tubulopathy with an estimated prevalence of 1–16 in 40,000 (refs. ^8–10^). Although hypocalciuria and hypomagnesemia in the presence of persistent renal salt wasting are landmark findings for GS in the differential diagnosis of chronic hypokalemia, genetic testing is still the gold standard to confirm GS^11–13^.

The *SLC12A3* gene on chromosome 16 is composed of 26 exons with >130 kb. To date, approximately 500 different mutations have been identified with a wide distribution along 26 exons and some introns of *SLC12A3* (refs. ^14–16^). Direct Sanger sequencing has long been regarded as the gold standard to detect the genetic mutations for GS. However, large genomic rearrangement, large deletion, and deep intronic mutations that were not so rare in GS may not be detected by direct sequencing^17^. It has been described that recurrent *SLC12A3* mutations were highly prevalent among unrelated GS families^18–20^. In a large French cohort of patients with GS, seven recurrent *SLC12A3* mutations (p.A313V, p.G394fs (c.1180+1G>T), p.G741R, p.L559P, p.R861C, p.H916fs (c.2883+1G>T), and p.C994Y) contributed to 41.5% of all identified allelic mutations^21^. We collected a large cohort of GS families^5,14^ and found that recurrent *SLC12A3* mutations occurred frequently with undefined prevalence. The rapid detection of these recurrent NCC mutations, albeit challenging, may provide an early genetic diagnosis of GS.

This study was aimed to define the prevalence of recurrent *SLC12A3* mutations from 130 genetically confirmed GS families and design a recurrent mutation-based detection plate with validation using TaqMan probe-based real-time polymerase chain reaction (RT-PCR). In addition, we clinically evaluated the feasibility of the diagnostic plate in newly diagnosed GS patients. The results to be reported indicated that 22 recurrent mutations of the 57 identified *SLC12A3* mutations comprised 87.1% of all allelic mutations. Using this simple recurrent mutation-based detection plate in 12 clinically diagnosed GS patients, 92.0% of allelic mutations were rapidly detected.

## Results

### Identification of *SLC12A3* mutations

**Fig. 1:**
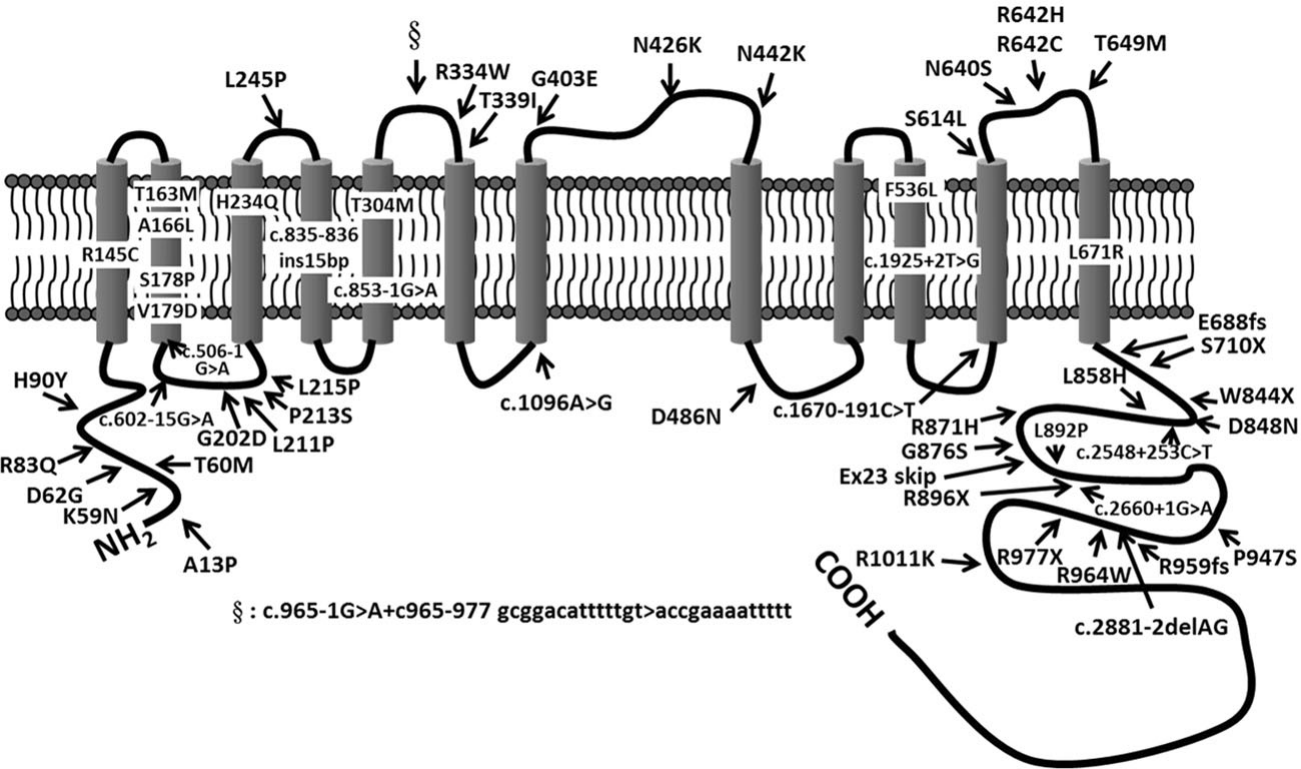
Topology of human NCC with position of each mutation in this study. Amino acid exchanges of the mutations identified in this study are shown and their locations are indicated by arrows.

As shown in Fig. 1, 57 different *SLC12A3* mutations including 10 novel ones in 130 unrelated GS families were identified. The most common mutation type was a missense mutation (47.5%), followed by frameshift (17.6%), splicing site mutations (17.3%), nonsense mutations (12.6%), and indels (5.0%). The majority of GS patients (112/130, 86.2%) were compound heterozygous. Eighteen GS families (13.8%) had triple mutations with p.T163M and p.R871H being exclusively on one allele at a much higher frequency (66.7%), suggestive of a founder effect (Table 1).

**Table 1:** Triple pathogenic *SLC12A3* mutations in 18 unrelated GS families.

Families	Mutation 1	Mutation 2	Mutation 3
01	p.T163M	p.R871H	p.N426K
02	p.T163M	p.R871H	p.R959fs
03	p.T163M	p.R871H	c.2548+253C>T
04	p.T163M	p.R871H	c.2548+253C>T
05	p.T163M	p.R871H	c.2660+1G>A
06	p.T163M	p.R871H	p.K59N
07	p.T163M	p.R871H	p.L245P
08	p.T163M	p.R871H	p.R977X
09	p.T163M	p.R871H	p.S178P
10	p.T163M	p.R871H	p.S710X
11	p.T163M	p.R871H	p.S710X
12	p.T163M	p.R871H	p.S710X
13	p.S710X	c.1096-2A>G	c.1096-2A>G
14	p.S710X	c.1670-191C>T	c.1670-191C>T
15	p.R83Q	p.S710X	p.W844X
16	p.H90Y	p.N640S	p.S710X
17	p.T60M	p.T60M	p.R959fs
18	p.A13P	p.T60M	p.R83Q

### Prevalence of recurrent *SLC12A3* mutations

**Fig. 2:**
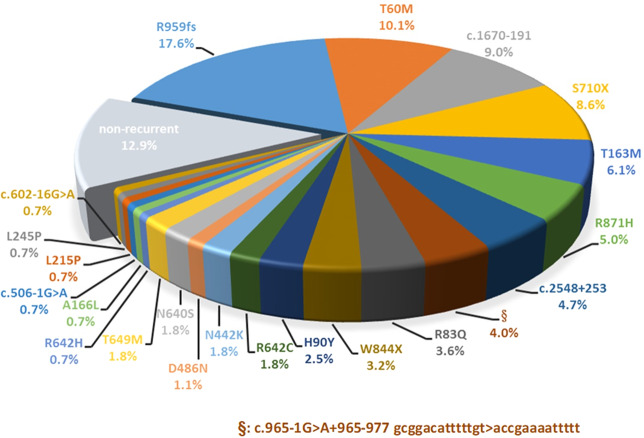
Twenty-two recurrent mutations with different percentage of all 57 *SLC12A3* mutations. Twenty two recurrent mutations comprise 87.1% of all allelic mutations, compared with 35 non-recurrent mutations responsible for only 12.9% of all allelic mutations.

As shown in Fig. 2, 22 recurrent mutations accounted for 87.1% of all allelic mutations (242 of 278 allelic mutations). All GS patients carried at least one recurrent mutation. The most prevalent mutations in order were p.R959fs, p.T60M, c.1670-191C>T, p.S710X, p.T163M, and p.R871H, responsible for 56.5% of the total allelic mutations. Of note, two deep intronic mutations (c.1670-191C>T in intron 13 and c.2548+253C>T in intron 21) that were undetected by conventional genetic tests constituted 13.7% of all allelic mutations.

### Characteristics of recurrent *SLC12A3* mutations

These 22 recurrent *SLC12A3* mutations were located in extracellular loops (7/22), intracellular loops (5/22), C-terminal domain (5/22), N-terminal domain (3/22), and rarely in transmembrane domains (2/22). A mutation (p.T60M) at the phosphorylation site of NCC regulated by STE20/SPS1-related proline/alanine-rich kinase/oxidative stress-responsive kinase-1 (SPAK/OSR1)^22^ was not only frequent in our cohort but also in Japan, Korea, and western countries^18,23,24^. Three recurrent mutations with p.D486N, p.R642C, and c.506-1G>A were reported in many different geographic areas^18,23–25^. Twelve recurrent mutations (p.R83Q, p.T163M, p.L215P, p.N442K, p.R642H, p.T649M, p.W844X, p.R871H, p.R959fs, c.602-16G>A, c.1670-191C>T, and c.2548+253C>T) have been reported in Japan^18,26,27^. Three recurrent mutations (p.T649M, p.W844X, and c.602-16G>A) were detected in western countries^25,28,29^ Mutation p.H90Y could be found in Korea^24^.

### Validation of recurrent mutation-based mutational detection plate

**Fig. 3:**
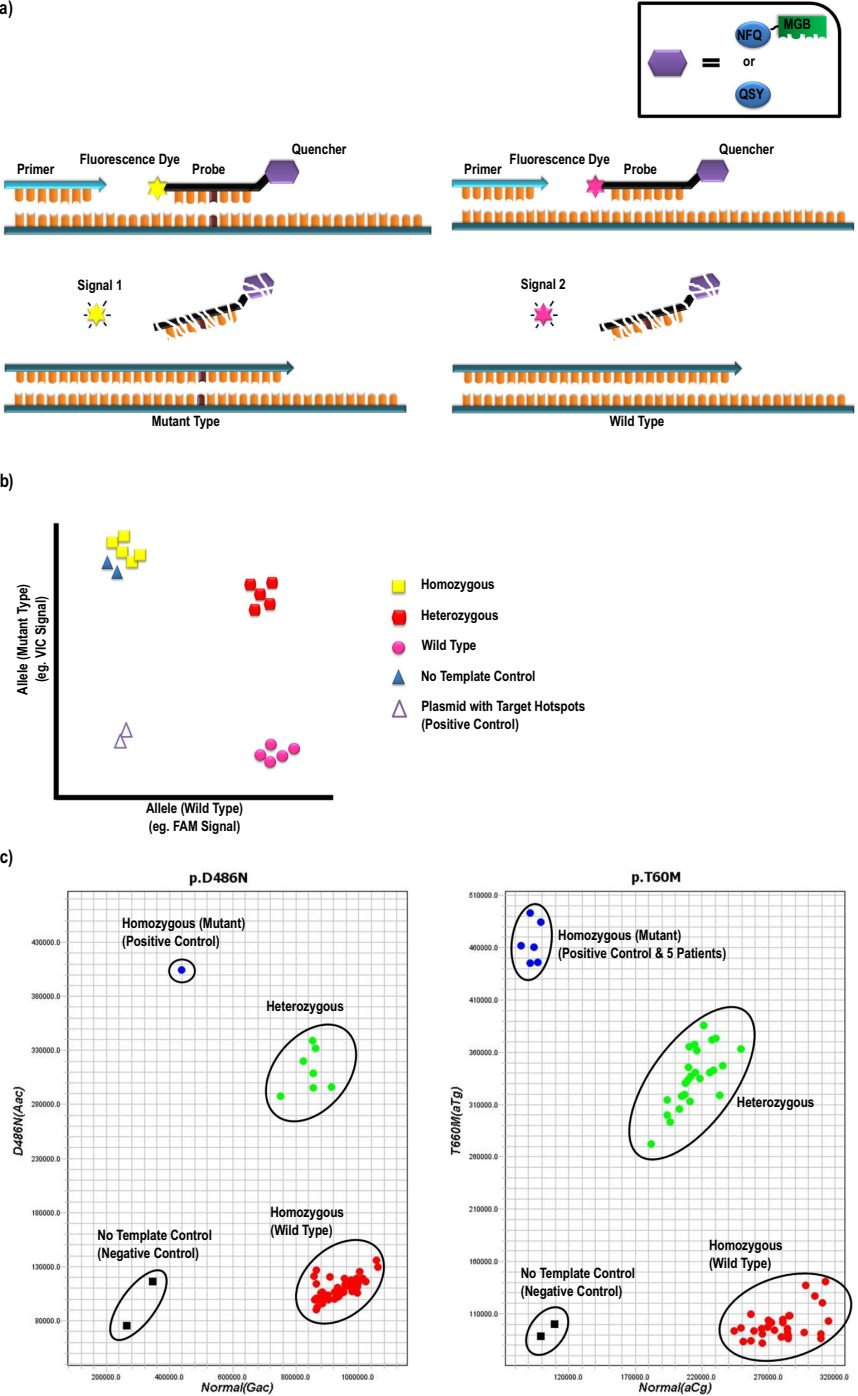
A schematic presentation of TaqMan probe-based RT-PCR and interpreration of allelic discrimination plot. **a** The probe is labeled with a 3′ quencher (a dark QSY or a non-fluorescent minor groove binder (MGB) quencher) and a 5′ fluorescent reporter dye (ABY, JUN, FAM, or VIC). During the standard procedure of RT-PCR, the TaqMan polymerase with 5′–3′ nuclease activity will release the fluorescent reporter which will be detected in each cycle. **b** Example of the output of allelic discrimination software and interpretation of allelic discrimination plot based on the signal measurement of VIC and FAM fluorescent reporter dye. **c** Interpretation of the results from our p.D486N and p.T60M assays on validation. The target recurrent single-nucleotide variants were inserted into vector pUC57 as positive control for each assay as well as two no-template controls as negative control. As shown with p.T60M, six blue plots represented one positive control and five homozygous samples. The number of green plots and red plots indicated the number of samples carrying one (heterozygous) or no (wild type) allelic mutation p.T60M.

As shown in Fig. 3, the mutation detection plate employing TaqMan probe-based RT-PCR for 22 recurrent *SLC12A3* mutations was created successfully with 100% accuracy. Every assay validated in samples from 130 GS patients and 50 healthy volunteers exhibited fairly high sensitivity (>99%) and specificity (>99%). One healthy volunteer harboring one recurrent mutation also confirmed by Sanger sequencing suggested a higher prevalence of GS carriers in Taiwan’s population.

### Clinical validation in newly diagnosed GS patients

As shown in Table 2 and Supplementary Fig. 2, 12 newly diagnosed GS patients were included for the detection of recurrent *SLC12A3* mutations. Ten (83.3%) of them were identified as biallelic recurrent mutations including one triple mutations. With direct sequencing, the remaining two GS patients with one recurrent mutation were confirmed to carry a novel splicing site mutation c.2285+2T>C (IVS18+2T>C) and p.D62G on the other allele, respectively. Overall, recurrent mutations were recognized in 92.0% (23/25) of allelic mutations.

**Table 2:** Recurrent mutation-based detection in clinically diagnosed GS patients.

No	Sex	Age	[K^+^]	[Mg^2+^]	U_Ca/Cr_	Allele 1	Allele 2
Unit		Yeard	mmol/L	mg/dl	mmol/mmol		
01	F	63	2.9	1.3	0.03	p.R959fs	p.R959fs
02	F	35	2.1	1.6	0.08	p.R642C	p.T649M
03	F	24	3.0	1.7	0.02	p.T60M	p.N442K
04	M	36	1.7	1.6	0.06	p.W844X	p.R959fs
05	M	24	3.2	1.7	0.08	p.R83Q	p.R871H
06	M	19	1.7	1.9	0.03	p.T60M	p.T60M
07	F	32	2.8	1.4	0.02	p.R83Q	**c.2285+2T>C**^**a**^
08	M	60	1.8	1.1	0.10	p.R83Q	p.R83Q
09	M	22	2.8	1.3	0.07	c.1670-191C>T	p.D486N
10	F	26	2.7	1.2	0.03	p.N442K	p.R959fs
11	F	28	2.4	1.3	0.14	p.R959fs	**p.D62G**^**a**^
12.	M	18	1.9	1.5	0.02	p.T163M/p.R871H	p.D486N

^a^The mutations detected by direct sequencing.

The two bold values represent mutations which were initially not detected by recurrent mutation-based mutation detection plate because both of them were not included in the recurrent mutations defined in this study. Since only monoallelic mutation was identified in the two patients, other genetic tests were conducted and the two SLC12A3 mutations were identified.

## Discussion

In this study, we identified 57 distinct *SLC12A3* mutations with 22 being recurrent in 130 unrelated genetically confirmed Taiwan GS families. The prevalence of these recurrent *SLC12A3* mutations was much higher, approximately 87.1% of all allelic mutations. Using TaqMan probe-based RT-PCR, we designed a novel recurrent mutation-based *SLC12A3* mutation detection plate that was successfully validated in GS patients and healthy subjects. In the prospective evaluation for 12 newly diagnosed GS patients, recurrent mutations were rapidly identified in 92.0% of all allelic mutations.

GS is one of the most common inherited salt-losing tubulopathies and genetic testing remains the gold standard to confirm the diagnosis^11–13^. With advanced molecular techniques, approximately 500 *SLC12A3* mutations have been reported in >1300 GS patients^14–16^. Compound heterozygosity with different mutations on each allele is the most common (up to 70%) with missense mutation being the most frequent type^30,31^. In our cohort, the prevalence of compound heterozygosity was even higher, approximately 86.2% of GS families. Of note, triple *SLC12A3* mutations with one mutation in one allele and two in the other have been increasingly reported^28,32^. We found that 13.8% of our GS families had triple *SLC12A3* mutations and all the mutations were classified as pathogenic according to the ACMG guidelines. Mutations p.T163M and p.R871H exclusively on the same allele in GS patients with triple mutations at a higher frequency suggest linkage disequilibrium secondary to a possible founder effect. The highly prevalent triple *SLC12A3* mutations indicate that a complete genetic analysis of family pedigree should still be warranted even though two pathogenic mutations were identified. Besides, this implies that there may be many mutational hotspots on *SLC12A3*; screening these hotspots will provide a rapid tool in the molecular diagnosis of GS.

Our cohort also showed a significantly higher occurrence of deep mutations within introns 13 and 21, resulting in a pseudo-exon inclusion in GS families^33^. To date, deep intronic mutations—usually missed by conventional sequencing analyses—have been recognized as causative variants in >75 diseases such as β-thalassemia but were rarely reported in GS patients previously^34^. The higher prevalence may highlight the importance of searching for deep intronic mutations using cDNA analysis in GS patients if first-line methodologies fail to identify causative mutations.

The meticulous analyses of all reported *SLC12A3* mutations to date clearly shows that recurrent mutations in *SLC12A3* were relatively common^5,24,35–37^. In Japan, two recurrent mutations (p.R642C and p.L858H) are reportedly responsible for 38.6% of all allelic mutations in GS families^37^. One European survey enrolling 164 independent families reported that 47 recurrent mutations out of 115 mutations accounted for 77.9% of all allelic mutations^23^. In this study, 22 recurrent mutations accounted for >87.1% *SLC12A3* allelic mutations. In light of the higher prevalence of recurrent mutations in *SLC12A3*, rapidly identifying them may be very helpful in the early molecular diagnosis of GS.

Routine genetic screening for *SLC12A3* mutations is still time-, labor-, and cost-consuming. Several time-saving methods have been developed to detect recurrent mutations in human-inherited diseases or cancer such as neurofibromatosis, epileptic disease, and breast cancer^13,38^. Allelic-specific TaqMan probe-based RT-PCR is a high-throughput method and has been widely used in virological testing, somatic mutation detection in cancers, and polymorphism screening^5,39,40^. Its use in identifying recurrent *SLC12A3* mutations in GS has not been evaluated. Using this methodology, we successfully created a mutation detection plate and confirmed its fairly high sensitivity and specificity. All mutations of validation samples, including deep intronic mutations, were accurately recognized with easily interpreted results.

A prospective test of this mutation detection plate in 12 newly diagnosed GS patients rapidly identified >90% of allelic mutations. For samples with single or without detectable recurrent mutations, we performed a step-by-step approach and identified two additional non-recurrent *SLC12A3* mutations on the other allele in two GS patients. Considering that the majority of recurrent mutations in our plate are recognized frequently in different geographic regions, we suggested that using the mutation detection plate as the primary screen for GS should improve the efficacy of GS diagnosis. Furthermore, this recurrent mutation-based detection approach may also be applied to other genetic diseases with a high prevalence of recurrent mutations such as hemophilia A, cystic fibrosis, and bilateral sensorineural hearing loss^41,42^.

Compared to other high-throughput platforms, our detection plate does not need to run cases in batches. The cost of our mutation detection plate is around 30 USD/patient with a rapid turnaround time (within 4 h), much cheaper and efficient than either targeted panel sequencing, which has a high cost (around 350 USD/patient) and slower turnaround time (5–7 days), or wholeexome sequencing (WES), which has a higher cost, far longer turnaround time (weeks), and requires dedicated professionals. Furthermore, mutations deep in the introns responsible for about 20% of mutant alleles will be missed in WES.

This study has some limitations. First, rare, novel, and non-recurrent mutations could not be recognized by this recurrent mutation-based detection plate. To expand the application, more mutational spots should be added to enhance the diagnostic efficiency. Second, we did not compare the cost-effectiveness between this mutation detection plate and conventionally used sequencing or other genetic analyses.

In conclusion, recurrent mutations in the *SLC12A3* gene are highly prevalent in Taiwan GS patients and can be quickly detected by this accurate recurrent mutation-based detection plate. Early detection of *SCL12A3* mutations may enhance the medical care quality in GS. Further investigation and validation is still warranted in other cohorts of GS.

## Methods

### Study design and participants

The study protocol was approved by the Institutional Review Board (B202005082) at Tri-Service General Hospital, National Defense Medical Center, Taipei, Taiwan. Prior written informed consent was obtained from all subjects and parental assent for any minors from their family members in our study according to the guidelines approved by the ethics vommittee.

In total, 130 unrelated families of 161 probands with genetically confirmed GS were consecutively enrolled over the past 15 years. There were 103 families with one proband, 23 families with two probands, and four families with three probands. Complete genetic analysis of the probands and family members was performed for cosegregation whenever available and also in all GS families with triple *SLC12A3* mutations. The enrolled families were not only identified from our hospital but also referred from other hospitals in different regions of Taiwan, including indigenous and Han populations^43–46^. All of them have persistent renal salt wasting, chronic hypokalemic metabolic alkalosis with renal K^+^ wasting (urine K^+^/Cr ratio >2 mmol/mmol), renal Mg^2+^ wasting with or without hypomagnesemia, and hypocalciuria or normocalciuria.

### Detection of pathogenic mutations in *SLC12A3*

Using genomic DNA and/or cDNA from blood leukocytes (National Center for Biotechnology Information No. NM-000339), a series of meticulous genetic analyses of *SLC12A3* were performed. Genetically confirmed GS was defined as recognition of inactivating *SLC12A3* mutations in both alleles. Sanger sequencing was performed first with primers pairs that were designed for exons and introns based on previous studies^6,33,47^. For those with no biallelic mutations, denatured high-performance liquid chromatography, direct sequencing, multiplex ligation-dependent probe amplification for large deletions, cDNA analysis via RT-PCR for splicing site and deep intronic mutations, and targeted sequencing panel for all genes relevant in the differential diagnosis of GS were performed in order as previously reported^14,33,47^.

To confirm the pathogenicity of the identified nucleotide variants, we excluded single-nucleotide variants with a frequency of >1% at first using a publicly available database (HapMap, dbSNP150 (http://www.ncbi.nlm.nih.gov/projects/SNP/), the 1,000 Genomes Project (http://www.1000genomes.org), the Genome Aggregation Database (gnomAD, https://gnomad.broadinstitute.org), and the Exome Aggregation Consortium (ExAC) database). Pathogenicity score then was evaluated via in silico computational methods using SIFT, PolyPhen2, LRT, Mutation Taster, Mutation Accessor, FATHMM, M-CAP, CADD, and GERP^38,48–50^. Furthermore, the pathogenicity of all candidate variants was annotated based on American College of Medical Genetics and Genomics (ACMG) standards and guidelines^51^.

### Definition of recurrent *SLC12A3* mutations and prevalence

Recurrent *SLC12A3* mutation was defined as the reappearance of the same mutation in two or more unrelated families. Triple mutations were defined as two different mutations in one allele and one mutation in another allele. If two or more siblings from one family carried the same mutations, “one occurrence” of each mutation was calculated to prevent the overestimation of prevalence. Accordingly, the prevalence of recurrent mutations was determined as the total number of recurrent mutations divided by the total number of all allelic mutations in 130 “families” rather than patients.

### Development of recurrent mutation-based mutation detection plate

To create a recurrent mutation-based detection plate, *SLC12A3* mutations that fulfilled the definition of recurrent mutation were obtained. Primers and TaqMan probes were designed by Primer Express software (Applied Biosystems, USA). Two types of dual-labeled probes were applied for RT-PCR in this study. The QSY probes incorporate a dark 3′ QSY quencher and a 5′ fluorescent reporter dye (ABY or JUN). The minor groove binder (MGB) probes contain a non-fluorescent quencher (NFQ) attached to a 3′ MGB moiety and a fluorescent reporter dye (FAM or VIC) incorporated into the 5′ end. Each mutation detection assay included a pair of primers and two allele-specific probes with different reporter dyes corresponding to mutant and wild-type alleles, respectively. Table 3 shows the primer and probe sequences.

**Table 3:** Sequences of primers and probes used for TaqMan probe-based RT-PCR.

Nucleotide	Protein	Mutation type	Forward primer (sense)	Reverse primer (antisense)	wild probe (Allele 1)	Mutatnt probe (Allele 2)
c.179C>T	p.T60M	Missense	GCATGCGCACCTTTGGC	TGCTGTTGGCATAGTGCTCATAT	CCTTTGGCTACAACAcGATCG	CACCTTTGGCTACAACAtGATC
c.248G>A	p.R83Q	Missense	CGCACCTTTGGCTACAACAC	CTTCTGAGACAGCACTTACCTTG	CTGGCTGACCTGcACTCC	CACTGGCTGACCTGtACTC
c.268G>A	p.H90Y	Missense	TGAGCCCCGGAAGGTCCG	CAGGGAAGTGGCCAGTCTTC	CACTGGCTGACCTGcACTC	CTGGCTGACCTGtACTCC
c.488C>T	p.T163M	Missense	CCAGATTCGTTGCATGCTCAAC	CAAGCCCTCCCTCCTCTTCC	CCCTGGATTAcGGCCCAG	CTGCCCTGGATTAtGGCC
c.496_497delGCinsCT	p.A166L	Missense	CCAGATTCGTTGCATGCTCAA	CAAGCCCTCCCTCCTCTTCC	CGGCCCAGgcAGGCATC	ATTACGGCCCAGctAGGCATC
c.506-1G>A	IVS3 as -1G>A	Exon skipping	GAAATGCCCTGCCTAAGCTTTG	ACCGACAGCAGGATGATGATC	TGCCCCCTGCAgTCCTGA	TGCCCCCTGCAaTCCTGA
c.602-16G>A	IVS4 as -16G>A	Exon skipping	CCAGCCAACCGACTCATCTG	GACTCCGGGAGATGAGGAAGTA	TCATGGTTCCCgGCTCTGCC	TTCATGGTTCCCaGCTCTGCC
c.644T>C	p.L215P	Missense	CCAGCCAACCGACTCATCT	GGCATTGGCGAAAGCGAAAA	CGATGGAGCCCCCAaGCTCT	ATGGAGCCCCCAgGCTCTG
c.734T>C	p.L245P	Missense	CGGTGGGCTTTGCAGAGA	GGGCGGAGGAGAGTGCAAT	CGTGCGGGACCtGCTCCAG	TGCGGGACCcGCTCCAG
c.965-1G>A+c965-977 gcggacatttttgt>accgaaaattttt		Exon7-8skipping	CCTTACTCATCAGGCCTTGCT	CCAGTCAGGCACCAAGTTCT	CCAgcggacatttttgTCCA	CCAaccgaaaattttTCCAG
c.1297C>G	p.N442K	Missense	AGCACAGCTGCCACTACG	ACAAACTTCCAGGCCTGCAAG	CCACTACGGCCTCATCAAcTAT	CGGCCTCATCAACgTATTACCAG
c.1456G>A	p.D486N	Missense	TCCCAGCCTAAGGGTGAGTG	GAAGCCGATCAGTGGGTACAG	TTGCGAGgACCAGCTGTAC	CCTTTGCGAGaACCAGCT
c.1670-191C>T	IVS13 as-191C>T	90 bp pseud-oexon	TGGATGCTCCTGGTGAAATGC	TCCTGGGCTGGGTCTACAGG	CAGCCAAAGCAGGcGTG	CCAAAGCAGGtGTGTGATT
c.1919A>G	p.N640S	Missense	CTGGCCCTCAGCTACTCG	AGGGTGGAGCCATCACTGG	AAGACCACATCAAGAaCTACCGG	CCACATCAAGAgCTACCGGTGA
c.1924C>T	p.R642C	Missense	GGTACAGGCTGGCTCCTACA	CAGGGTGGAGCCATCACTG	GAACTACcGGTGAGCAGAGC	CAAGAACTACtGGTGAGCAG
c.1925G>A	p.R642H	Missense	GCTCCTCGGTACAGGCTGG	TCCCAGGGTGGAGCCATCA	CAAGAACTACCgGTGAGCAG	CAAGAACTACCaGTGAGCAG
c.1946C>T	p.T649M	Missense	TGGCTCCTGCCCTTTTCC	GCCGGGCGGAAGTTG	GTGCCTGGTGCTCAcGG	AGTGCCTGGTGCTCAtGG
c.2129C>A	p.S710X	Nonsense	TGGCTGAACAAGAGGAAGATCAAG	ATGGCACCTGCATGAGGATC	TCTACTcGGATGTCATTGCCG	AAGGCCTTCTACTaGGATGT
c.2532G>A	p.W844X	Nonsense	GCAGGGCAAGAAGACCATAG	GCAGGAGGGCTGATCCAAG	CTACTGgCTCTTTGACGATGG	CCATAGACATCTACTGaCTCTTTG
c.2548+253C>T	IVS21 ds +253 C>T	238 bp pseudo-exon	GTCGCCATGAGATGGAAACTTC	TCTTTCTATCTGTGACTCTATC	TTAGGGGCACAGGcAGGT	CTTAGGGGCACAGGtAGG
c.2612G>A	p.R871H	Missense	AGAGGAGGTGGAGCAAATGC	TCTCCTGGTCCATCCTGTTAATCT	AAGATCCgTGTGTTCGTAGGC	ATGCAAGATCCaTGTGTTCG
c.2875-2876delAG	p.R959fs	Frameshift	GACTGCCCCTGGAAGATCTCAG	GTCCCTGACCCAGTGATGTGT	CGAAGAACAGagTCAAGGTGC	CGAAGAACAGTCAAGGTGCA

Using a 96-well plate, all TaqMan probe-based RT-PCR experiment was performed in a 10 μL reaction mixture consisting of 5 μL 2× TaqMan master mix buffer (Life Technologies), 300 nM of each primer, 300 nM of probes, and 100 ng DNA template. The preparation of the mixtures was carried out on EzMate, an Automated Liquid Handling & Pipetting System. The TaqMan probe-based RT-PCR was done in QuantStudio 5 Real-Time PCR System (Thermo Fisher) with the following thermal cycle program: 60 °C for 30 s followed by 95 °C for 5 min and then 40 cycles of 95 °C for 15 s and 60 °C for 1 min. Real-time data were collected during 40 cycles of amplification and were analyzed using the TaqMan Genotyper software v.1.3 (Life Technologies).

### Validation of all recurrent mutations

To test the function of each mutation detection assay in this recurrent mutation-based mutation detection plate, a blind study was conducted for each assay. DNA samples from all 130 unrelated GS families were included with concealed genotypes and plated in duplicates on the 96-well plates, using 100 ng DNA of each sample as a template in the above-described RT-PCR assay. The genotypes of each sample were uncovered for comparison at the end of this blind study to evaluate the accuracy. Besides, target recurrent mutations were inserted into vector pUC57 as a positive control for each assay as well as two no-template controls as negative controls. When referring to the sensitivity and specificity, each TaqMan assay was accessed in DNA samples including GS patients with or without specific mutations and healthy subjects.

### Clinical validation in newly diagnosed GS patients

Patients newly diagnosed as GS over a year were prospectively enrolled to validate this mutation detection plate with Sanger sequencing confirmation. Vector pUC57 carrying homozygous and heterozygous target recurrent mutations was applied concomitantly as a positive control. DNA samples from three healthy subjects and vector pUC57 with wild-type *SLC12A3* were also used as wild-type control as well as one no-template control as a negative control (Supplementary Fig. 1). If the detection plate fails to make a definite diagnosis, further genetic analyses as mentioned above were performed.

### Reporting summary

Further information on research design is available in the Nature Research Reporting Summary linked to this article.

## Supplementary information

Supplementary Information

Reporting Summary

## Data Availability

Data generated or analyzed during this study are included in this published article and its supplementary information files.
