# Supplementary material for: Allele-specific RT-PCR for the rapid detection of recurrent SLC12A3 mutations for Gitelman syndrome
Source: NPJ Genom Med. 2021 Aug 13;6:68. doi: 10.1038/s41525-021-00230-8 (PMC8363728; doi:10.1038/s41525-021-00230-8)
Supplement: Supplementary file 1 — Supplementary Information [file 41525_2021_230_MOESM1_ESM.pdf]

Supplementary Information

Table of content

Supplementary Figure 1. Example and interpretation of allelic discrimination using the hotspot-based TaqMan assay in newly-diagnosed GS patients.

Supplementary Figure 2. Interpretation of validation in 12 newly-diagnosed GS patients.

Supplementary Figure 1. Example and interpretation of allelic discrimination using the hotspot-based TaqMan assay in newly-diagnosed GS patients

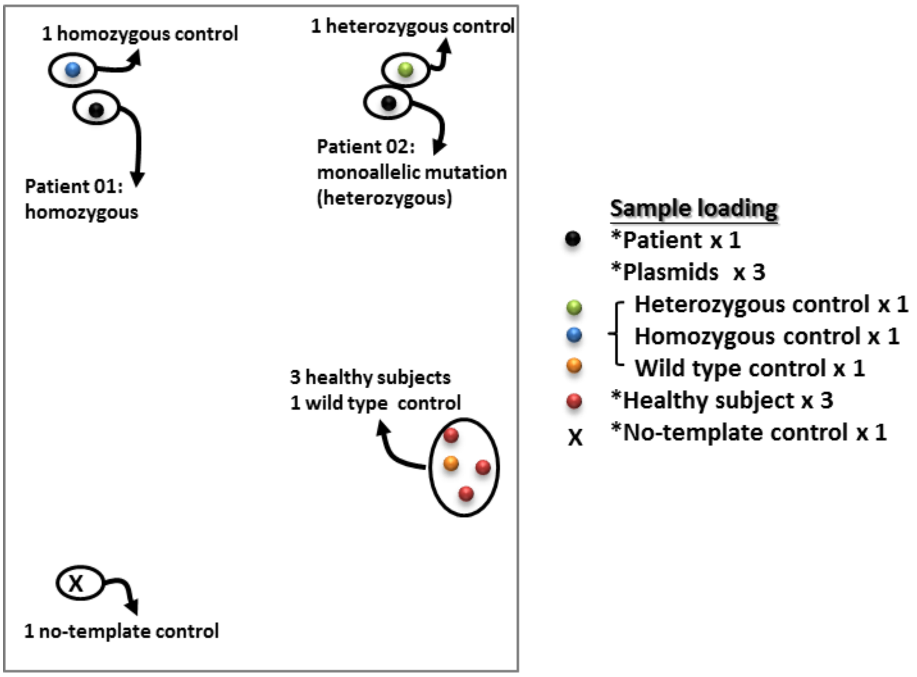

Supplementary Figure 2. Interpretation of validation in 12 newly-diagnosed GS patients.

Case 1

p.R959fs

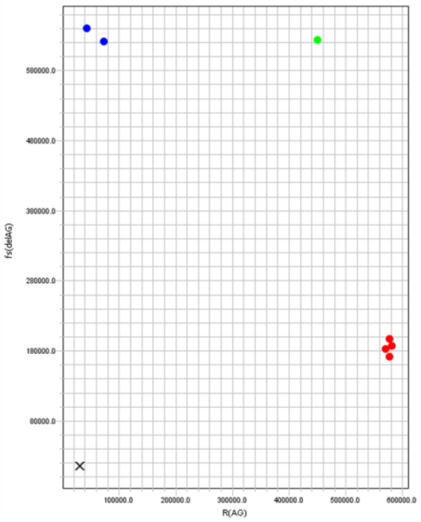

<u>**Diagnosis: homozygous p.R959fs**</u>

Case 2

p.R642C

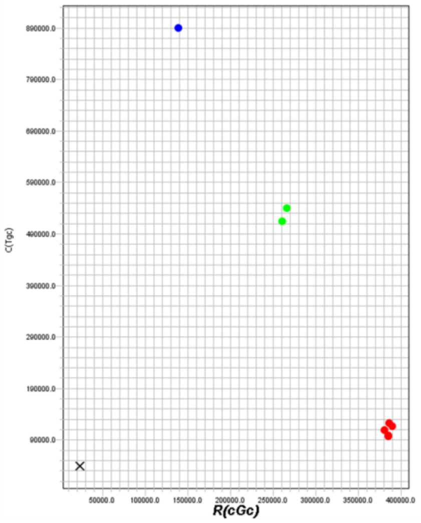

p.T649M

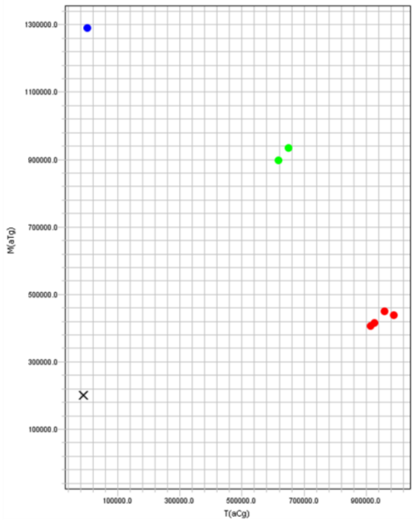

<u>**Diagnosis: heterozygous p.R642C/p.T649M**</u>

Case 3

p.T60M

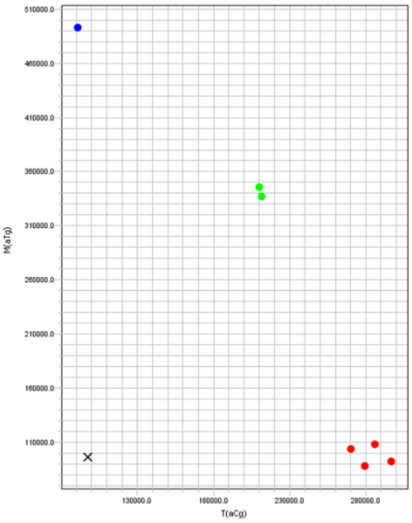

p.N442K

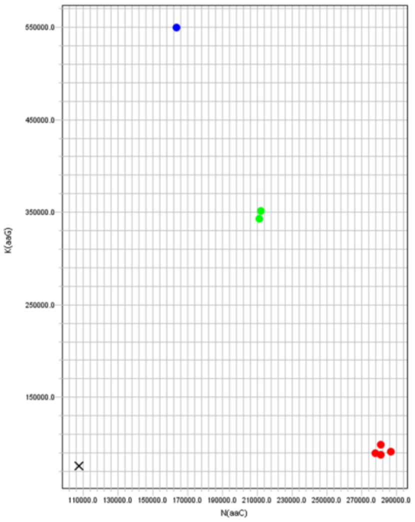

Diagnosis: heterozygous p.T60M/p.N442K

Case 4

p.W844X

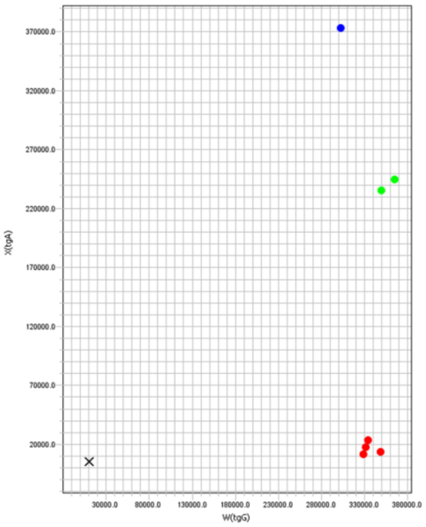

p.R959fs

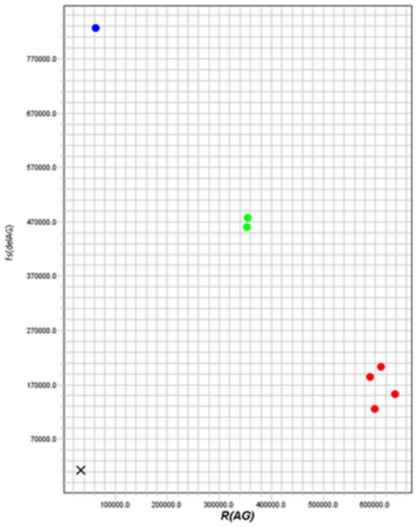

Diagnosis: heterozygous p.W844X/p.R959fs

Case 5

p.R83Q

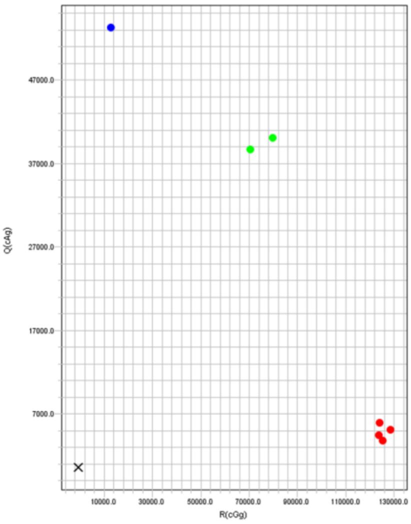

p.R871H

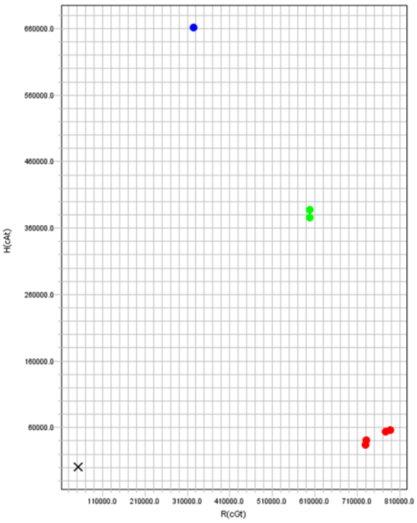

Diagnosis: heterozygous p.R83Q/p.R971H

Case 6

p.T60M

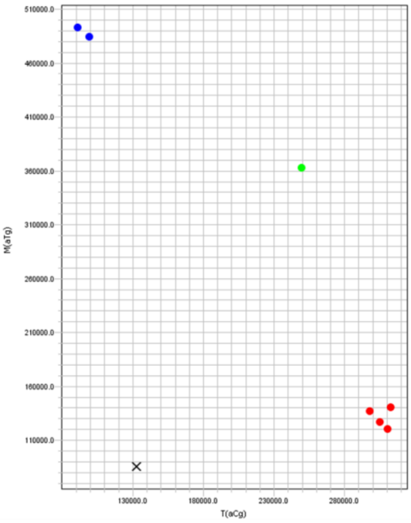

Diagnosis: homozygous p.T60M

Case 7

p.R83Q

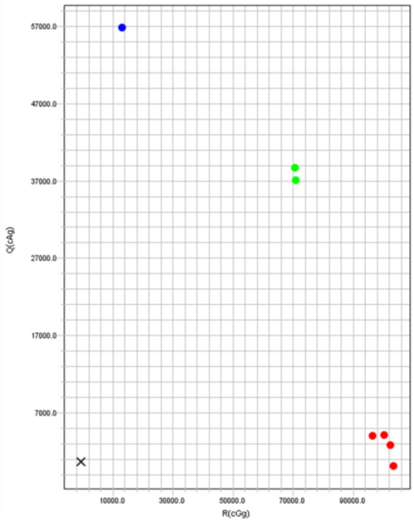

Diagnosis: monoallelic p.R83Q

Case 8

p.R83Q

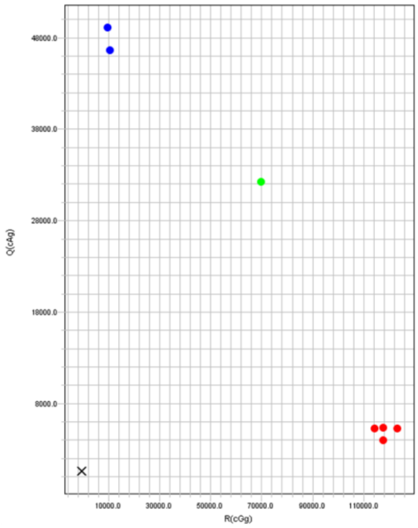

Diagnosis: homozygous p.R83Q

Case 9

p.D486N

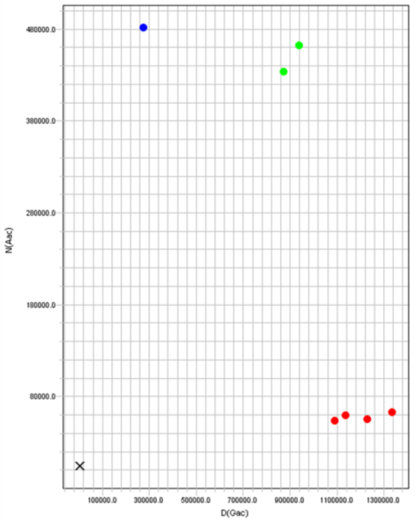

c1670-191C>T

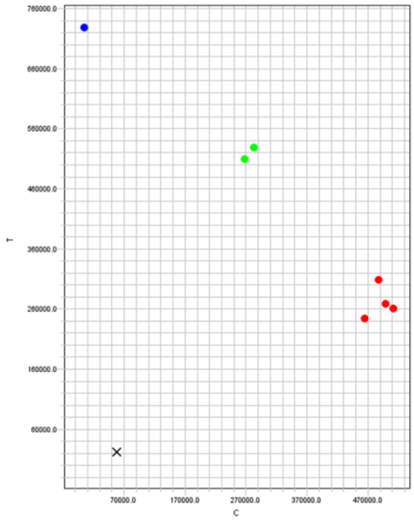

Diagnosis: heterozygous p.D486N/c1670-191C>T

Case 10

p.N442K

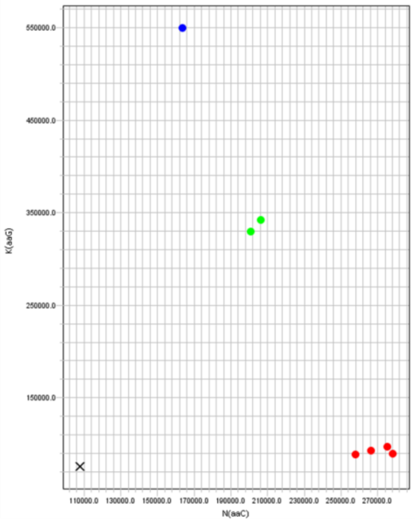

p.R959fs

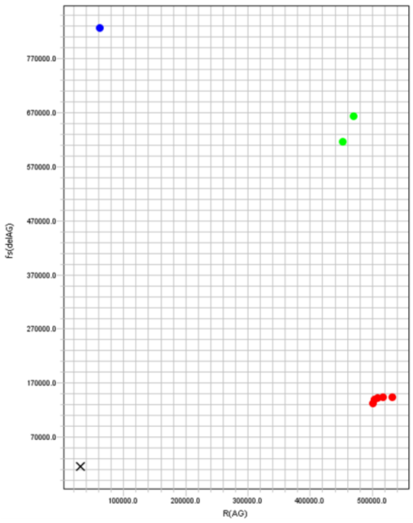

Diagnosis: heterozygous p.N442K/p.R959fs

Case 11

p.R959fs

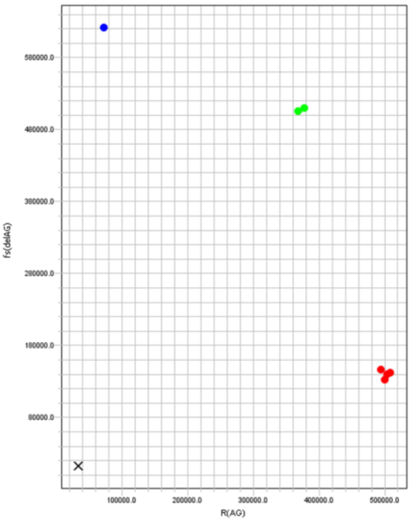

Diagnosis: monoallelic p.R959fs

Case 12

T163M

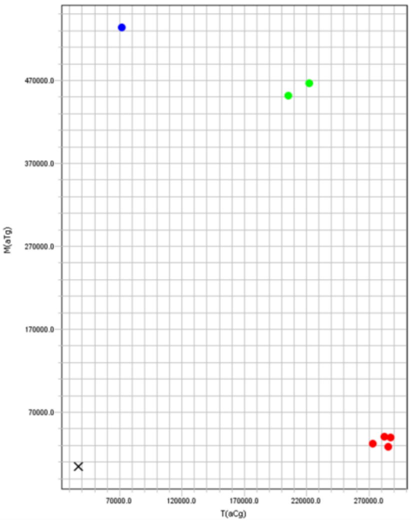

R871H

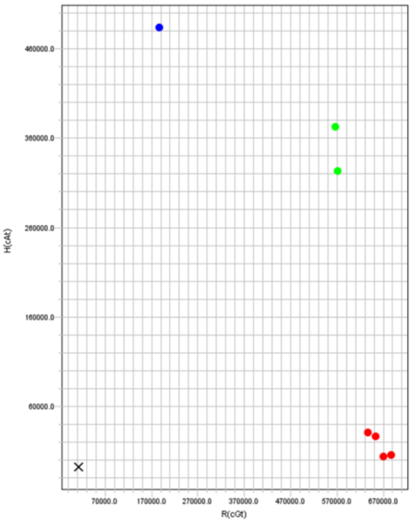

D486N

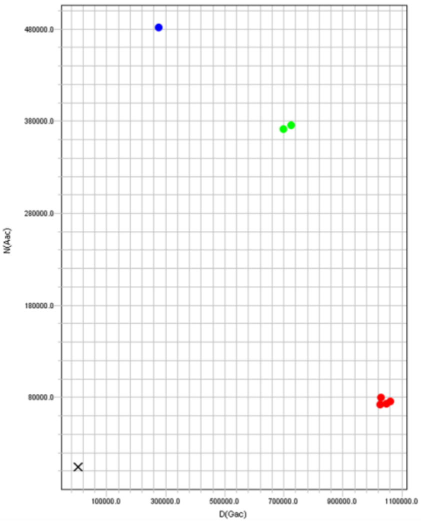

Diagnosis: triple p.T163M/p.R871H/p.D486N
