# Supplementary material for: Allele-specific RT-PCR for the rapid detection of recurrent SLC12A3 mutations for Gitelman syndrome
Source: NPJ Genom Med. 2021 Aug 13;6:68. doi: 10.1038/s41525-021-00230-8 (PMC8363728; doi:10.1038/s41525-021-00230-8)
Supplement: Supplementary file 2 — Reporting Summary [file 41525_2021_230_MOESM2_ESM.pdf]

nature portfolio

Corresponding author(s): Shih-Hua Lin

Last updated by author(s): Jul 11, 2021

Reporting Summary

Nature Portfolio wishes to improve the reproducibility of the work that we publish. This form provides structure for consistency and transparency in reporting. For further information on Nature Portfolio policies, see our Editorial Policies and the Editorial Policy Checklist.

Statistics

For all statistical analyses, confirm that the following items are present in the figure legend, table legend, main text, or Methods section.

n/a	Confirmed	
☐	☒	The exact sample size (n) for each experimental group/condition, given as a discrete number and unit of measurement
☐	☒	A statement on whether measurements were taken from distinct samples or whether the same sample was measured repeatedly
☒	☐	The statistical test(s) used AND whether they are one- or two-sided<br>*Only common tests should be described solely by name; describe more complex techniques in the Methods section.*
☒	☐	A description of all covariates tested
☒	☐	A description of any assumptions or corrections, such as tests of normality and adjustment for multiple comparisons
☒	☐	A full description of the statistical parameters including central tendency (e.g. means) or other basic estimates (e.g. regression coefficient) AND variation (e.g. standard deviation) or associated estimates of uncertainty (e.g. confidence intervals)
☒	☐	For null hypothesis testing, the test statistic (e.g. F, t, r) with confidence intervals, effect sizes, degrees of freedom and P value noted<br>*Give P values as exact values whenever suitable.*
☒	☐	For Bayesian analysis, information on the choice of priors and Markov chain Monte Carlo settings
☒	☐	For hierarchical and complex designs, identification of the appropriate level for tests and full reporting of outcomes
☒	☐	Estimates of effect sizes (e.g. Cohen's d, Pearson's r), indicating how they were calculated

Our web collection on statistics for biologists contains articles on many of the points above.

Software and code

Policy information about availability of computer code

Data collection	n/a
Data analysis	n/a

For manuscripts utilizing custom algorithms or software that are central to the research but not yet described in published literature, software must be made available to editors and reviewers. We strongly encourage code deposition in a community repository (e.g. GitHub). See the Nature Portfolio guidelines for submitting code & software for further information.

Data

Policy information about availability of data

All manuscripts must include a data availability statement. This statement should provide the following information, where applicable:

- Accession codes, unique identifiers, or web links for publicly available datasets
- A description of any restrictions on data availability
- For clinical datasets or third party data, please ensure that the statement adheres to our policy

Data generated or analyzed during this study are included in this published article and its supplementary information files.

Field-specific reporting

Please select the one below that is the best fit for your research. If you are not sure, read the appropriate sections before making your selection.

☒ Life sciences ☐ Behavioural & social sciences ☐ Ecological, evolutionary & environmental sciences

For a reference copy of the document with all sections, see nature.com/documents/nr-reporting-summary-flat.pdf

Life sciences study design

All studies must disclose on these points even when the disclosure is negative.

Sample size	In total, 130 unrelated families of 161 probands with genetically confirmed GS were consecutively enrolled over the past 15 years. When referring to the sensitivity and specificity, 50 healthy volunteers were recruited except for the 130 GS families. In clinical validation, 12 newly diagnosed GS patients were enrolled.
Data exclusions	n/a
Replication	DNA samples in this study were plated in duplicate on the detection plate. All methodologies to detect the mutations in the SLC12A3 gene including Sanger sequencing, denatured high-performance liquid chromatography (DHPLC), multiplex ligation-dependent probe amplification (MLPA), cDNA analysis via RT-PCR and targeted sequencing panel for all genes relevant in the differential diagnosis of GS were performed twice at least to confirm the reproducibility of the results of pathogenic variants.
Randomization	n/a
Blinding	n/a

Reporting for specific materials, systems and methods

We require information from authors about some types of materials, experimental systems and methods used in many studies. Here, indicate whether each material, system or method listed is relevant to your study. If you are not sure if a list item applies to your research, read the appropriate section before selecting a response.

Materials & experimental systems

n/a	Involved in the study	
☒	☐	Antibodies
☒	☐	Eukaryotic cell lines
☒	☐	Palaeontology and archaeology
☒	☐	Animals and other organisms
☐	☒	Human research participants
☒	☐	Clinical data
☒	☐	Dual use research of concern

Methods

n/a	Involved in the study	
☒	☐	ChIP-seq
☒	☐	Flow cytometry
☒	☐	MRI-based neuroimaging

Human research participants

Policy information about studies involving human research participants

Population characteristics	In total, 130 unrelated families of 161 probands with genetically confirmed GS were consecutively enrolled over the past 15 years from hospitals in different regions of Taiwan, including indigenous and Han populations. All if them have persistent renal salt wasting, chronic hypokalemic metabolic alkalosis with renal K+ wasting (urine K+/Cr ratio > 2 mmol/mmol), renal Mg2+ wasting with or without hypomagnesemia, and hypocalciuria or normocalciuria. In clinical validation, 12 newly diagnosed GS patients were enrolled with above-described clinical and laboratory features as the 130 GS families. Besides, 50 healthy volunteers without either clinical and laboratory features or biallelically pathogenic variants of GS were enrolled.
Recruitment	Participants with GS were enrolled from hospitals in different regions of Taiwan, including indigenous and Han Populations.
Ethics oversight	The study protocol was approved by the Institutional Review Board (B202005082) at Tri-Service General Hospital, National Defense Medical Center, Taipei, Taiwan. Prior written informed consent from was obtained from all subjects and parental assent from minors in our study according to the guideline approved by Ethics Committee.

Note that full information on the approval of the study protocol must also be provided in the manuscript.
